# Comparison Between Facilitating and Suppressing Facial Emotional Expressions Using Frontal EEG Asymmetry

**DOI:** 10.3389/fnbeh.2020.554147

**Published:** 2020-10-09

**Authors:** Hiromichi Takehara, Shigekazu Ishihara, Tatsuya Iwaki

**Affiliations:** ^1^Graduate School of Medical Technology and Health Welfare Science, Hiroshima International University, Higashihiroshima, Japan; ^2^Department of Assistive Rehabilitation, Hiroshima International University, Higashihiroshima, Japan; ^3^Department of Psychology, Komazawa University, Setagaya, Japan

**Keywords:** emotion, frontal EEG asymmetry, the motivational direction model, facial control, laterality index, microstate analysis

## Abstract

The prefrontal cortex plays a key role in emotional state. Electroencephalography (EEG) studies have reported relationships between frontal asymmetry in the alpha band, emotional state, and emotion-related motivation. The current study investigated whether the positive or negative valence of emotional stimulation or the behavioral intention to either facilitate or suppress one’s facial expression in response to these stimuli is reflected in relevant changes in frontal EEG asymmetry. EEG was recorded while participants either produced a facial expression that was in accord with positive or negative feelings corresponding to image stimuli, or suppressed their facial expressions. The laterality index of frontal alpha power indicated greater relative right frontal activity while participants suppressed facial expression compared with facilitating facial expression during emotional stimulation. However, there was no difference in frontal asymmetry between the presentation of image stimuli showing facial expressions corresponding to positive vs. negative emotions. These results suggested that frontal asymmetry was related to the control of facial emotional expressions rather than the perception of positive vs. negative emotions. Moreover, microstate analysis revealed that the appearance rate of microstate class B with polarity in the left frontal area increased during the suppression of facial expressions. The present results suggested that frontal asymmetry reflects the control of facial emotional expressions, which supports the motivational direction model.

## Introduction

In recent years, with technological progress in machine learning and measurement instruments, new techniques for estimating emotion have been developed. Psychophysiological studies estimating emotion are employed in various fields, such as neuromarketing and the development of communication tools for people with physical disabilities.

To examine the neural mechanisms of emotion, brain function imaging devices such as functional magnetic resonance imaging (fMRI) and positron emission tomography (PET) have been widely used to measure whole brain activity. However, electroencephalography (EEG) has also been used in psychophysiological studies because EEG measurement involves a relatively small burden for participants. In addition, EEG has high temporal resolution and, with the development of analytical technology, is now capable of examining cortical neuronal networks. In particular, the relationship between frontal EEG asymmetry and emotional state has been investigated for a relatively long time.

Frontal EEG asymmetry is assumed to be present when there are differences between the left and right frontal regions in alpha band (8–13 Hz) power. Higher alpha band power is used as an index to indicate cortical activity suppression (Pfurtscheller et al., 1996; Klimesch, 1999; Coan and Allen, 2004). Activation of the frontal region is considered to be caused by decreased alpha band power values. Several studies have focused on alpha band power and examining the relationship between frontal asymmetry and emotional state. The affective valence model and the motivational direction model are subject to ongoing debate regarding whether frontal EEG asymmetry reflects emotional state or motivations related to approach–withdrawal behavior.

In studies of the affective valence model, based on the characteristics of alpha waves, it has been reported that relatively strong left frontal activity is related to positive emotions (approach), while relatively strong right frontal activity is related to negative emotions (withdrawal; e.g., Davidson, 1992, 1998). For example, previous studies have used video clips (Davidson and Fox, 1982; Jones and Fox, 1992) and music (Schmidt and Trainor, 2001) as emotional stimuli to elicit emotional responses in participants while frontal EEG asymmetry was measured. The results revealed that positive stimuli, such as joy and happiness, elicited greater relative left frontal activity, whereas negative stimuli, such as fear and sadness, elicited greater relative right frontal activity. These findings support the affective valence model.

Conversely, the motivational direction model predicts that relative frontal asymmetry reflects motivational direction. Motivational direction distinguishes approach motivation to move toward a stimulus from withdrawal motivation to move away from a stimulus (Harmon-Jones et al., 2013). In many cases, the affective valence model and the motivational direction model predict the same result because positive emotions are associated with approach and negative emotions are associated with withdrawal. In other words, the experimental results described above can be explained not only by the affective valence model but also by the motivational direction model.

“Anger” has often been focused on in experiments investigating which of the two models is correct, because the predictions of the two models differ for anger. In the affective valence model, anger would be expected to be associated with greater relative right frontal activity as a negative emotion, whereas, in the motivational direction model, anger would be expected to be associated with greater relative left frontal activity, as an approach motivation. Previous studies have reported that anger is associated with greater relative left frontal activity, which supports the motivational direction model rather than the affective valence model (Harmon-Jones, 2004; Hewig et al., 2004).

Papousek et al. (2018) examined motivational direction using anger-related stimuli. In one study, the researchers investigated the relationship between motivational direction and personality traits using two sound stimuli: angry aggression (approach) and desperate crying (withdrawal). The results revealed that participants with higher levels of antagonism exhibited EEG responses indicating greater relative activation related to approach and less relative activation related to withdrawal (Papousek et al., 2018). Conversely, participants with higher levels of detachment showed EEG responses indicating more relative activation related to withdrawal and less relative activation related to approach. Thus, relative frontal asymmetry reflected personality traits as motivational direction rather than just reflecting anger or sadness. This finding indicated that the two models can be compared by focusing on motivational direction for emotions other than anger.

Papousek et al. (2018) interpreted their results in a social–emotional context. Similarly, in the current study, we paid attention to emotional control in communicative situations. In social life, people sometimes exhibit facial expressions that are contrary to their actual feelings, such as forced smiles. These facial expressions are important for positive communication and in clinical applications. In the current study, we focused on the control of facial emotional expressions as motivational direction.

For example, Ekman and Davidson (1993) instructed participants to produce specific emotional facial expressions while frontal asymmetry was measured. The results revealed that genuine smiles of joy were associated with greater relative left frontal activity compared with non-genuine smiles of joy (Ekman and Davidson, 1993). In another study, participants produced facial expressions corresponding to high approach positive emotions, facial expressions corresponding to low approach positive emotion, or a neutral facial expression, while frontal asymmetry was compared between each facial expression condition (Price et al., 2013). The results revealed that participants who produced facial expressions corresponding to high approach positive emotion exhibited greater relative left frontal activity than participants in the other two facial expression conditions (Price et al., 2013). These findings indicated that high approach positive emotion caused greater relative left frontal activity compared with low approach positive emotion, in accord with the motivational direction model.

These previous findings suggest that facial emotional expression is related to motivational direction. This is inconsistent with the classical facial feedback hypothesis (Tomkins, 1962), which suggests that facial expression affects emotion. If facial expression control regulates motivational direction rather than emotion, it would be expected to have a substantial effect on application such as stress coping strategies. However, the relationship between facial control and frontal asymmetry has not yet been fully elucidated. In particular, it remains unclear whether suppressing facial expression inhibits emotion or facilitates withdrawal behavior. In a previous study using suppression of facial expression, suppression of facial expression during watching sad and amusing film clips decreased subjective rating scores of amusement for both film types, compared with a condition involving no suppression (Gross and Levenson, 1997). Thus, the suppression of facial emotional expression may be related to withdrawal motivation.

In the current study, we directly compared affective valence using affective images (positive vs. negative) and motivational direction using the control of facial emotional expressions (facilitation vs. suppression).

Based on the two models discussed above, we sought to test the following two hypotheses: (1) if PFC asymmetry reflects the perception of positive and negative emotional states, based on the affective valence model, relatively greater activity of the left compared with right PFC would be expected following stimulation with positive compared with negative images; and (2) if PFC asymmetry reflects the control of facial emotional expressions of facilitation and suppression, based on the motivational direction model, relatively greater activity of right than left PFC would be expected following emotional stimulation with instructions to suppress emotional expressions, regardless of the valence of the emotional stimuli.

In addition, EEG microstate analysis was performed in conjunction with typical alpha band power differences between bilateral frontal regions. The EEG microstate refers to a minimum unit during which a momentary electric field structure is obtained from the spatial distribution of multi-point EEG measurement and is classified based on the similarity of the electric field structure. Microstate analysis is a method of analyzing the characteristics of EEG microstates as sequential maps. It is assumed that each map or the sequence of each map is related to brain function and psychological activities (Lehman et al., 1987; Koenig et al., 2002). Characteristic EEG activity related to emotional state or the control of facial emotional expressions was investigated by comparing the appearance rate of microstate maps and the sequences of microstate maps between conditions.

## Materials and Methods

### Participants

In the current study, 25 undergraduate or graduate students (10 females, mean age 21.4 years, age range 19–26 years) participated. Participants reported their handedness based on the Edinburgh Handedness Inventory, and all were right-handed. All participants had normal or corrected-to-normal vision, and no participants reported any neurological or psychiatric problems. Written informed consent was obtained from all participants prior to participation in the experiment. All experimental procedures were approved by the ethics committee of Hiroshima International University (No. 17-022).

### Stimuli

Before the experiment, 150 images (positive, neutral, or negative; 50 images each) were used from the International Affective Picture System (IAPS; Lang et al., 1999) and the Open Affective Standardized Image Set (OASIS; Benedek et al., 2017). Twenty undergraduate participants rated the images for valence and arousal scores. Next, 90 images (positive, neutral, or negative; 30 images each) were selected based on the rating scores (see Table 1). These 90 images were used as image stimuli in this study. The content of the positive images included smiling children, puppies, and similar subjects and was intended to elicit positive emotions, such as happiness. The content of negative images included the scene of a robbery, an unsanitary toilet, and an accident scene and was intended to elicit negative emotions, such as disgust and fear. Neutral images were objects and landscapes, such as electric outlets, memo pads, and crosswalks.

**Table 1:** Rating scores for affective image stimuli.

	Positive		Neutral		Negative	
	Mean	SD	Mean	SD	Mean	SD
Valence	7.32	0.44	5.06	0.24	2.25	0.37
Arousal	4.10	1.10	4.39	0.63	5.79	0.57

### Facial Expression Conditions

A facial expression task was used to manipulate the control of participants’ facial emotional expressions while the image stimuli were presented. The facial expression task had two conditions: facilitation and suppression. In the facilitation condition, the participant was instructed to produce a facial expression that was in accord with each image stimulus (positive or negative). In the suppression condition, the participant was instructed to not change their facial expression, regardless of the image stimulus being presented. Both conditions were performed for all participants.

### Procedure

**Figure 1:**
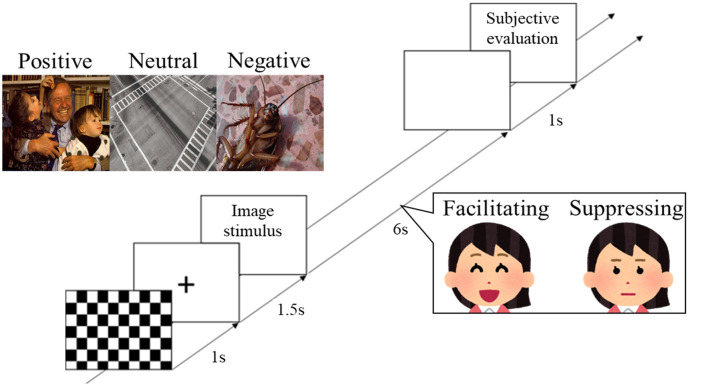
Protocol for stimulus presentation and facial expression responses. The three labeled images show examples of the image stimuli from international affective picture system (IAPS) and open affective standardized image set (OASIS) that were presented in the experiment (positive: IAPS, 2340; neutral: OASIS, Crosswalk 1; negative: OASIS, Cockroach 1). The two images of the woman show the facial expression task that was performed by participants during presentation of the image stimuli.

During the experiments, participants sat in a reclining chair in an electromagnetically shielded room. Two facial expression conditions were presented in a counterbalanced order. At the beginning of each condition, EEG was recorded for 2 min with eyes closed, and 2 min with eyes open. Next, participants performed the task session. First, a checkered pattern was presented for 1 s, a fixation point was displayed for 1.5 s, and the image stimulus was then presented for 6 s (Figure 1). During image presentation, the participant performed facial control according to the experimental condition. After a blank screen was presented for 1 s, subjective rating of the image stimuli was performed (see “Measurements” section). This trial was repeated for all 90 image stimuli. The image stimuli were presented in a random order at a visual angle of 11.64° on a 20-inch LCD monitor (2007FPb, Dell Inc., Round Rock, TX, USA) approximately 1.5 m in front of the participants. E-prime 2.0 (Psychology Software Tools, Sharpsburg, PA, USA) was used to control the experiments.

### Measurements

Participants evaluated the image stimuli by rating them on a nine-point scale for the following three items: positive–negative (valence), excited–calm (arousal), and elicited facial expression—did not elicit facial expression (facial expression strength). In addition, the emotions elicited by the stimuli were selected from six categories: sadness, anger, happiness, disgust, fear, and neutral.

### EEG and Electromyography (EMG) Recordings

EEG and electromyography (EMG) were continuously recorded throughout the experiment using a Neurofax device (EEG-1100, Nihon Kohden, Tokyo, Japan). EEG was sampled at a rate of 500 Hz and amplification of 0.05–120 Hz. The 25 electrodes were placed on Fpz, Fp1, Fp2, Fz, F3, F4, F7, F8, FC1, FC2, FC5, FC6, Cz, C3, C4, T7, T8, Pz, P3, P4, P7, P8, Oz, O1, and O2, according to the International 10-20 system. EEG was recorded from 25 scalp sites using an EEG cap with Ag–AgCl electrodes and was re-referenced from the average of earlobe measurements. Electrode impedances were below 10 kΩ.

EMG was sampled at a rate of 500 Hz and amplification of 5–250 Hz. The electrodes were attached to the right sides of the face over each corrugator supercilii (CS) and zygomatic major (ZM) muscle region. EMG of the CS and ZM was recorded using the same filter as the EEG and was used as an index during the facial expression task.

### EEG and EMG Analyses

EEG data analysis was performed with independent component analysis (ICA) using EEGLAB 15 (Delorme and Makeig, 2004) in the MATLAB platform (2017b; MathWorks, Natick, MA, USA). Independent components that were considered to arise from eye blinks, and movement artifacts were removed. For EEG data after ICA, epochs were discarded if they included extreme amplitude values (exceeding −100 or +100 μV). From a total of 30 trials, the average numbers of trials for each condition after removing invalid components and epochs were as follows. The facilitating facial expression condition had an average of 29.64 trials for the positive condition, 29.72 for the neutral condition, and 29.68 for the negative condition. The suppressing facial expression condition had an average of 29.48 trials for the positive condition, 29.52 for the neutral condition, and 29.68 for the negative condition. The following preprocessed EEG data were analyzed following EEG data collection.

Spectral analysis: The power spectrum of each site was computed for every 6 s (one trial) of EEG data using a fast Fourier transform (FFT) for 25 scalp sites. The Hamming window was used for smoothing; the window size was 2 s, and the overlap was 50%. For the computed power spectrum of the alpha band (alpha power), the standard deviation (SD) was calculated for each participant, and any epochs that included extreme alpha power (exceeding three SD) were removed from further analysis. For the corresponding right and left regions, the laterality index was calculated using the following formula: (left side alpha power − right side alpha power)/(left side alpha power + right side alpha power) × 100. Greater alpha band power was used as an index of the suppression of cortical activity; positive values on the laterality index indicate greater relative right regional activity, while negative values on the laterality index indicate greater relative left regional activity.

EEG microstate analysis: EEG microstate analysis was performed using Cartool (Brunet et al., 2011). First, an alpha band filter was applied to all EEG waveforms, and the data were downsampled to 100 Hz. One epoch was a 6 s segment, and the global field power (GFP) at the local maxima was detected. EEG topographies were obtained from detected GFP peaks. Cluster analysis was performed using the *k*-means clustering algorithm, and EEG topographies were clustered. After examining the number of clusters, the EEG topography, segmented by cluster, was labeled. Finally, the appearance rate of microstate classes and the sequence of microstate classes were computed.

The EMG data were analyzed to confirm whether the participant made a facial expression consistent with the experimental condition. EMG amplitude was averaged with 6 s epochs while presenting image stimuli for each condition. After EMG amplitude rectification, mean EMG amplitude values of 3,000 points data for 6 s were calculated for each trial. These amplitudes were averaged for each image stimulus and facial expression condition in the CS and ZM regions.

### Statistical Analysis

Two-way repeated-measures analysis of variance (ANOVA) was used to compare the subjective evaluation of image stimuli, EMG activity, and the laterality index, and *post hoc* testing was then performed using the Bonferroni correction. The Greenhouse–Geisser correction was used to adjust the degrees of freedom. The appearance rates of microstate classes computed by microstate analysis were compared using the Wilcoxon signed-rank test and nonparametric statistics. A paired *t*-test was also performed to compare the doublet sequence of map patterns in the microstate analysis. Statistical significance was defined as *p* < 0.05 for all analyses. Analyses were performed using SPSS 24.0 (IBM, Armonk, NY, USA).

## Results

### Subjective Ratings

**Figure 2:**
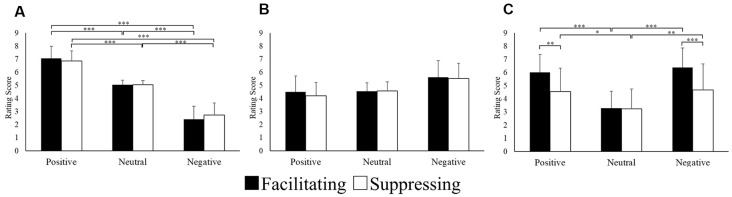
Mean subjective rating scores for valence **(A)**, arousal **(B)**, and facial expression strength **(C)**. *p < 0.05, **p < 0.01 and ***p < 0.001.

Data for the subjective rating scores were analyzed using two-way repeated ANOVA with 2 levels of control (facilitation vs. suppression) × 3 levels of emotion (positive vs. neutral vs. negative). Figure 2 shows the results of the subjective rating scores for the image stimuli. For the valence scores, there was a significant interaction (*F*_(1.17,28.30)_ = 5.45, *p* = 0.022, $\eta_{\text{p}}^{2}$ = 0.18, *ε* = 0.59). After *post hoc* testing, there was a significant difference between positive, neutral, and negative images in the two facial expression conditions (*p* < 0.001). For the arousal rating score, there was a main effect of the emotion condition (*F*_(1.22,29.45)_ = 9.25, *p* = 0.003, $\eta_{\text{p}}^{2}$ = 0.27, *ε* = 0.61). *Post hoc* testing revealed that the scores for negative images were significantly higher than those for positive (*p* < 0.05) or neutral (*p* < 0.001) images. The interaction was significant for facial expression strength (*F*_(1.60,38.51)_ = 28.57, *p* = 0.000, $\eta_{\text{p}}^{2}$ = 0.54, *ε* = 0.80). After *post hoc* testing, there was a significant difference for positive (*p* < 0.01) and negative (*p* < 0.001) images between the two facial expression conditions. Furthermore, in the facilitating facial expression condition, scores of facial expressions for positive (*p* < 0.001) and negative (*p* < 0.001) images were significantly higher than that those for neutral images. Similarly, in the suppressing facial expression condition, the scores of facial expressions for positive (*p* < 0.05) and negative (*p* < 0.01) images were significantly higher than those for neutral images.

**Figure 3:**
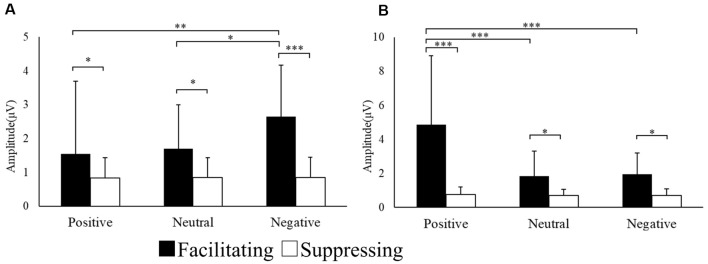
Mean electromyography (EMG) amplitude for CS **(A)** and ZM activity **(B)** in each condition. *p < 0.05, **p < 0.01 and ***p < 0.001.

In addition to the subjective ratings, the EMG responses of the CS and ZM were analyzed using two-way repeated ANOVA. Figure 3 shows the mean amplitude of EMG responses for each region. The interaction was significant for the EMG amplitude of the CS (*F*_(1.21,29.11)_ = 6.94, *p* = 0.009, $\eta_{\text{p}}^{2}$ = 0.22, ɛ = 0.60). With *post hoc* testing, there was a significant difference between the two facial expression conditions for positive (*p* < 0.001), neutral (*p* < 0.05), and negative (*p* < 0.05) images. Moreover, the CS EMG amplitude for negative images was significantly greater than those for positive (*p* < 0.01) and neutral (*p* < 0.05) images in the facilitating facial expression condition. The interaction was significant in the EMG amplitude of the ZM (*F*_(1.07,25.69)_ = 21.14, *p* = 0.000, $\eta_{\text{p}}^{2}$ = 0.46, ɛ = 0.53). After *post hoc* testing, there was a significant difference between the two facial expression conditions for positive (*p* < 0.001), neutral (*p* < 0.05), and negative (*p* < 0.05) images. In addition, in the facilitating facial expression condition, the ZM EMG amplitude for positive images was significantly greater than that for neutral (*p* < 0.001) and negative (*p* < 0.001) images.

### Laterality Index

**Figure 4:**
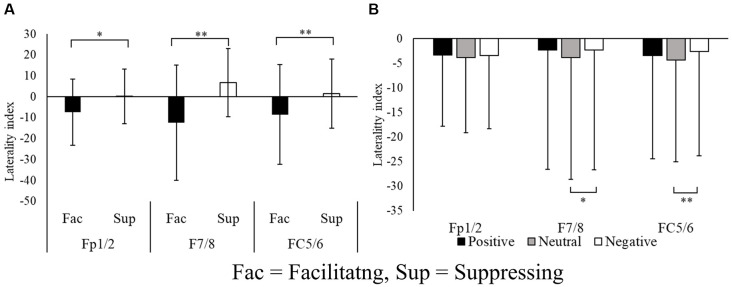
Laterality indices of the pairs of electrode sites at the frontal region **(A)** and for each image stimulus **(B)**. *p < 0.05, **p < 0.01.

The laterality index, which was calculated from the alpha power, was examined using pairs of electrode sites in the frontal region (Fp1/2, F3/4, F7/8, FC1/2, FC5/6). A two-way repeated ANOVA with 2 levels of control (facilitation vs. suppression) × 3 levels of emotion (positive vs. neutral vs. negative) was performed five times (one for each pairs of electrode sites) for the laterality index. Figure 4 shows the laterality index of sites exhibiting significant differences. The laterality index of F3/4 and FC1/2 did not show any significant differences. In contrast, Fp1/2, F7/8, and FC5/6 all exhibited significant differences in the laterality index. The main effect of the facial expression condition was significant at Fp1/2 (*F*_(1.00,24.00)_ = 4.31, *p* = 0.048, $\eta_{\text{p}}^{2}$ = 0.15, ɛ = 1.00), F7/8 (*F*_(1.00,24.00)_ = 12.68, *p* = 0.001, $\eta_{\text{p}}^{2}$ = 0.34, ɛ = 1.00), and FC5/6 (*F*_(1.00,24.00)_ = 7.942, *p* = 0.009, $\eta_{\text{p}}^{2}$ = 0.24, ɛ = 1.00).

The main effect of emotion was significant at F7/8 (*F*_(1.72,41.48)_ = 5.39, *p* = 0.011, $\eta_{\text{p}}^{2}$ = 0.18, ɛ = 0.86) and FC5/6 (*F*_(1.81,43.47)_ = 6.72, *p* = 0.003, $\eta_{\text{p}}^{2}$ = 0.21, ɛ = 0.90). *Post hoc* testing revealed that the laterality index in the neutral condition had a larger negative value than that for negative images (*p* < 0.01). These results indicate that the facilitating facial expression condition was associated with greater relative left frontal activity, whereas the suppressing facial expression condition was associated with greater relative right frontal activity.

### EEG Microstate Analysis

**Figure 5:**
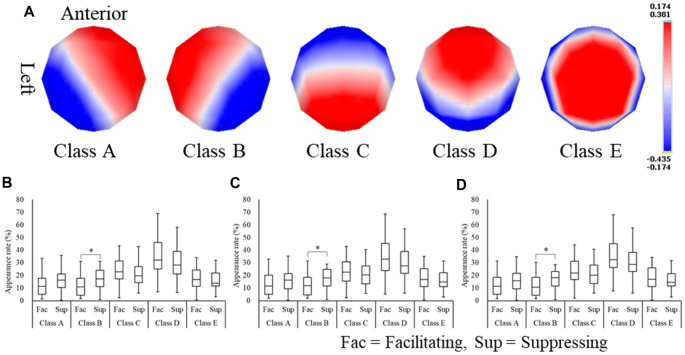
Microstate class maps **(A)** and the appearance rates of microstate classes for positive **(B)**, neutral **(C)**, and negative **(D)** images. *p < 0.05.

To examine EEG spatial characteristics in relation to emotional state, microstate analysis was applied to the EEG data for 6 s during image stimulus presentation. As a result, five microstate classes were extracted (Figure 5A) and were labeled (A–E). These five microstate classes were commonly found throughout the experimental conditions. Next, these five microstate classes were fitted to the EEG raw data, and the appearance rate of each microstate class in every experimental condition was computed (Figures 5B–D). We observed that the appearance rate of the microstate classes did not differ between the emotion conditions but differed between facial expression conditions. To analyze the difference in appearance rate between facial expression conditions, the Wilcoxon signed-rank test was performed. The appearance rate of microstate class B (from right occipital to left frontal areas) was significantly higher in positive (*Z*_(24)_ = −2.65, *p* = 0.008, *r* = −0.53), neutral (*Z*_(24)_ = −2.27, *p* = 0.023, *r* = −0.45), and negative (*Z*_(24)_ = −2.38, *p* = 0.017, *r* = −0.47) images in the suppressing facial expression condition compared with the facilitating facial expression condition.

Next, to examine the relationship between the order of the appearance pattern of microstate classes and emotional state, the numbers of doublet sequences of each microstate class were compared between the facilitating and suppressing facial expression conditions for each emotion using a paired *t*-test. The doublet sequences of the microstate classes were calculated as follows: the number of transitions from each microstate class to another microstate class, normalized by the fraction of the total number of microstate class transitions of participants. For example, the AA sequence means that microstate class A appears after microstate class A, and the AB sequence means that microstate class B appears after microstate class A. The BB sequence was significantly higher for positive (*t*_(24)_ = −3.43, *p* = 0.002, *r* = 0.57), neutral (*t*_(24)_ = −2.95, *p* = 0.007, *r* = 0.51), and negative (*t*_(24)_ = −3.14, *p* = 0.004, *r* = 0.54) images in the suppressing facial expression condition compared with the facilitating facial expression condition. Furthermore, the EB sequence was significantly higher for positive (*t*_(24)_ = −2.25, *p* = 0.034, *r* = 0.41) and negative (*t*_(24)_ = −2.09, *p* = 0.048, *r* = 0.39) images in the suppressing facial expression condition compared with the facilitating facial expression condition. In addition, the BE sequence was significantly higher for only positive (*t*_(24)_ = −2.07, *p* = 0.049, *r* = 0.38) images in the suppressing facial expression condition compared with the facilitating facial expression condition.

## Discussion

The present study directly compared affective valence and motivational direction in PFC asymmetry by manipulating the presentation of affective images (positive, neutral, and negative) and facial expression tasks (facilitating and suppressing facial emotional expressions). We hypothesized that PFC asymmetry would support: (1) the affective valence model if PFC asymmetry reflected the perception of emotional state, and relatively greater activity of the left than right PFC would be expected following stimulation with positive compared with negative images; or (2) the motivational direction model if PFC asymmetry reflected the control of facial emotional expressions, and relatively greater activity of the right than left PFC would be expected following emotional stimulation with an instruction to suppress emotional expressions.

First, the subjective ratings for the image stimuli were examined. In terms of valence, participants gave positive images higher scores and negative images lower scores, regardless of the facial expression conditions. Thus, it was confirmed that the image stimuli guided participants’ emotions appropriately in accord with the experimental conditions. For arousal, negative images were given higher scores than positive or neutral images. This result supports the confounding relationship with valence that has traditionally been accepted (Bradley et al., 2001). It is noteworthy that the level of arousal was higher in the negative condition than in the other conditions. However, the arousal rating scores were still relatively low, and there was no difference between the two facial expression conditions.

For positive and negative images, facial expression strength was given a higher score than for neutral images, and the scores for both image types were higher in the facilitating facial expression condition than in the suppressing facial expression condition. Regarding EMG responses, CS activity increased for negative images, while ZM activity increased for positive images. This result was consistent with previous reports that CS activity is increased in the presence of angry facial expressions, while ZM activity is increased in the presence of happy facial expressions (Winkielman et al., 2006; Achaibou et al., 2008; Cannon et al., 2010). Together, these results suggest the validity of the manipulation of emotion induction and facial control in the present study. Therefore, the current results suggest the involvement of an asymmetrical model, which is considered in more depth below.

We first examined the laterality index of the alpha power. In the laterality indices of Fp1/2, F7/8, and FC5/6, alpha power was greater in the left frontal area during suppression of facial expression compared with when facial emotional expression was facilitated during emotional stimulation. Because greater alpha power is used as an index to indicate suppression of cortical activity, these results indicate that greater relative left PFC activity was exhibited in the facilitating facial expression condition, and greater relative right PFC activity was exhibited in the suppressing facial expression condition. Regarding emotional stimulation, negative images elicited greater relative right PFC activity compared with neutral images, but not compared with positive images. Applying these results to the affective valence model, right PFC activity was consistent with negative emotional stimulation, but inconsistent with positive emotional stimulation. Considering the laterality index results, PFC asymmetry appeared to be caused by facial control rather than emotional state. This result supports the hypothesis that PFC asymmetry reflects the control of facial emotional expressions of facilitation and suppression, in accord with the motivational direction model.

Choi et al. (2016) analyzed frontal alpha asymmetry between the coping strategies for negative emotions. The results indicated that left frontal activity during reappraisal was relatively greater than that exhibited while simply viewing the negative images (control), but there was no difference in frontal asymmetry between the suppressing facial expression condition (suppression as coping strategy) and a control condition (Choi et al., 2016). This result is inconsistent with our findings of right frontal activity while suppressing facial expression. In the present study, the experimental conditions included suppressing facial expression for positive images as well as negative images. It is possible that suppressing facial expression for positive images is more strongly related to withdrawal behavior. If so, the positive suppression condition might have effectively induced right frontal activity while suppressing facial expression.

A large body of research indicates that context-dependent changes in EEG alpha asymmetry at F3/4 are highly sensitive to activation of relative approach vs. withdrawal motivation elicited by emotionally relevant situations or stimuli (Harmon-Jones and Gable, 2018). However, in the present study, frontal asymmetry was not found in F3/4 but was found in F7/8 in the ventrolateral prefrontal cortex (vlPFC). According to a previous brain imaging study, the vlPFC is involved in response selection and inhibition (Badre and Wagner, 2007). In addition, it has been reported that the vlPFC is activated by deliberate emotion regulation, including distraction and reappraisal (Ocshner et al., 2009; McRae et al., 2010). Considering these findings together, vlPFC activity in F7/8 might reflect deliberate emotion regulation related to approach vs. withdrawal motivation in the facial expression task. In addition to F7/F8, the frontal pole area (Fp1/2) showed frontal asymmetry between facial expression conditions. The frontal pole area is reported to be associated with the voluntary control of social emotional behavior (Volman et al., 2011a) and involved in the process of coordinating rapid action selection processes, emotional conflict detection, and inhibition of emotional responses (Volman et al., 2011b). In the present study, participants performed facilitating facial expressions, producing facial expressions according to their own emotional state and suppressing facial expressions that conflicted with their own emotional state. Thus, asymmetry in the frontal pole area appeared to reflect participants’ choice of facial control for emotional stimulation and/or responses when suppressing emotional facial expressions.

As mentioned above, the laterality index differed between the neutral and negative images, particularly in the ventrolateral frontal areas. Because the laterality indices had lower values for neutral images compared with negative images, the neutral condition exhibited greater relative left ventrolateral frontal area activity. Conversely, the negative emotion condition was associated with greater relative right ventrolateral frontal area activity. However, the laterality index values in the positive images were no different from those of other image types. Based on the affective valence model, the laterality index in the positive emotions would be expected to exhibit a relatively strong negative value (and, thus, greater relative left frontal area activity), but this was not shown in the results. A possible explanation for this contradiction may come from the subjective facial expression strength scores. The current results revealed that CS and ZM activity differed between the two facial expression conditions in all emotional stimulation conditions. However, unlike positive and negative images, the facial expression strength score in neutral images was no different between the two facial expression conditions. This suggests that participants did not subjectively feel the elicited facial expressions in the neutral image condition, although facial muscle activities differed between the two facial expression conditions. Therefore, differences in facial muscle activities between facilitating and suppressing facial expression of neutral images may be controlled by an unconscious process. It is possible that facial control comprises an unconscious control process and a deliberate control process. In other words, deliberate control of participants’ facial emotional expressions may not have been involved in processing neutral images. It was previously reported that the right inferior frontal cortex (Nakamura et al., 1999) and the right PFC (Gorno-Tempini et al., 2001) are activated when reading facial emotions. Although both of these studies used facial expression images, the greater relative right frontal area activity in negative images was caused by the perception of emotion elicited by controlling facial expressions. In contrast, because positive images are associated with approach motivation, relative left frontal activity would be expected in response to positive images. The right frontal activity mentioned above for facial control may have canceled out left frontal activity related to approach motivation, and, consequently, the laterality index for positive images may not have differed between neutral and negative images.

As a result of microstate analysis, five map patterns were obtained in the present study. Among these map patterns, microstate class A–D map patterns were similar to the map patterns that have been repeatedly confirmed in the eyes-closed resting state (Milz et al., 2017; Michel and Koenig, 2018). These four microstate classes were also found in different mental states, such as task (Milz et al., 2016) and sleep (Brodbeck et al., 2012) states. In the current study, microstate classes similar to these were also found, although the experimental conditions were different to those reported in previous studies.

Focusing on the appearance rate of each microstate class revealed differences between the facial expression conditions, but, similarly to the laterality index, there were no differences between the emotion conditions. Among the five microstate classes, microstate class B showed a difference in the appearance ratio, and this was significantly higher in the suppressing facial expression condition than the facilitating facial expression condition. That is, microstate analysis showed an increase in the appearance rate of microstate class B (from the right occipital to left frontal areas). This finding is consistent with the laterality index results. Therefore, our model supported the notion that (2) PFC asymmetry reflects the control of facial emotional expression of facilitation and suppression, based on the results of the microstate analysis.

Previous studies that identified the functional role of microstate classes using fMRI reported that microstate class B is related to visual processing, including the secondary visual cortex (BA18) and the visual association cortex (BA19; Damoiseaux et al., 2006; Mantini et al., 2007; Britz et al., 2010). In addition, activation of the right frontal region has been reported during response inhibition (e.g., in the Go/No-Go task). Goldin et al. (2008) suggested that an extensive network of brain regions is implicated in cognitive control (the PFC area) and visual–spatial processing (the precuneus and occipital areas) when inhibiting facial emotions in response to a negative image. These findings may be related to the increase in the appearance rate of microstate class B in the suppressing facial expression condition in the current study.

Next, in terms of the doublet sequence of the microstates, the BB sequence was increased for all image stimuli in the suppressing facial expression condition. This result was interpreted as indicating that the appearance rate of microstate class B was higher in the suppressing facial expression condition than in the facilitating facial expression condition. In addition, the BE sequence in the positive and negative images and the EB sequence in the positive images only were greater in the suppressing facial expression condition than in the facilitating facial expression condition. Although there was no evidence of a relationship between emotion or facial control and the specific sequences of microstate maps, the results suggested that a sequence from microstate classes B to E may be related to the suppression of emotion. This conclusion was supported by the greater appearance rate of microstate class B in the suppressing facial expression condition, and the finding that these sequences were different for positive and negative images in the suppressing facial expression condition.

In conclusion, the aim of the present study was to examine traditional PFC asymmetry models as the affective valence model and the motivational direction model by directly comparing the combination between affective images and the control of facial emotional expressions. The results suggest that PFC asymmetry reflects the control of facial emotional expressions, including facilitation and suppression, regardless of the emotional state. These findings supported the motivational direction model, which is currently the dominant model. However, it is possible that the sadness elicited by the affective images may have included anger, and this may have influenced the results of PFC asymmetry. PFC asymmetry was observed both in the laterality index and in the EEG microstate. However, no neutral condition for facial expressions was included in the current experimental design. If PFC asymmetry during facial expression was contrasted with an appropriate neutral condition, the activation of frontal asymmetry could be confirmed more clearly. In addition, in the current study, facial control was manipulated by instructing participants to change their facial expressions. Thus, the observation of asymmetry in the frontal region (F7/8 and FC5/6) might be related not only to the motivational direction but also to the function of producing facial expressions. Emotional regulation should therefore be investigated using a form of manipulation other than facial expression in future studies.

Furthermore, EEG microstate analysis suggested the existence of map sequence patterns that are related to positive and negative emotions during suppressing facial expression. Further research, including studies examining cognitive processing in the emotional state and emotional regulation, as well as studies focusing on temporal dynamics, are needed to construct a more robust frontal asymmetry model.

## Data Availability Statement

The raw data supporting the conclusions of this manuscript will be made available by the authors, without undue reservation, to any qualified researcher.

## Ethics Statement

The studies involving human participants were reviewed and approved by the ethics committee of Hiroshima International University. The patients/participants provided their written informed consent to participate in this study.

## Author Contributions

HT, SI, and TI contributed to the conception and design of the study. HT performed the EEG and statistical analysis and wrote the first draft of the manuscript. TI contributed to the interpretation of data and assisted in preparing the manuscript. All authors contributed to the article and approved the submitted version.

## Conflict of Interest

The authors declare that the research was conducted in the absence of any commercial or financial relationships that could be construed as a potential conflict of interest.
